# Syndecans as Modulators and Potential Pharmacological Targets in Cancer Progression

**DOI:** 10.3389/fonc.2014.00004

**Published:** 2014-02-03

**Authors:** Despoina Barbouri, Nikolaos Afratis, Chrisostomi Gialeli, Demitrios H. Vynios, Achilleas D. Theocharis, Nikos K. Karamanos

**Affiliations:** ^1^Biochemistry, Biochemical Analysis and Matrix Pathobiology Research Group, Laboratory of Biochemistry, Department of Chemistry, University of Patras, Patras, Greece

**Keywords:** proteoglycans, syndecans, shed syndecans, heparan sulfate, cancer, tumor microenvironment, pharmacological targeting

## Abstract

Extracellular matrix (ECM) components form a dynamic network of key importance for cell function and properties. Key macromolecules in this interplay are syndecans (SDCs), a family of transmembrane heparan sulfate proteoglycans (HSPGs). Specifically, heparan sulfate (HS) chains with their different sulfation pattern have the ability to interact with growth factors and their receptors in tumor microenvironment, promoting the activation of different signaling cascades that regulate tumor cell behavior. The affinity of HS chains with ligands is altered during malignant conditions because of the modification of chain sequence/sulfation pattern. Furthermore, matrix degradation enzymes derived from the tumor itself or the tumor microenvironment, like heparanase and matrix metalloproteinases, ADAM as well as ADAMTS are involved in the cleavage of SDCs ectodomain at the HS and protein core level, respectively. Such released soluble SDCs “shed SDCs” in the ECM interact in an autocrine or paracrine manner with the tumor or/and stromal cells. Shed SDCs, upon binding to several matrix effectors, such as growth factors, chemokines, and cytokines, have the ability to act as competitive inhibitors for membrane proteoglycans, and modulate the inflammatory microenvironment of cancer cells. It is notable that SDCs and their soluble counterparts may affect either the behavior of cancer cells and/or their microenvironment during cancer progression. The importance of these molecules has been highlighted since HSPGs have been proposed as prognostic markers of solid tumors and hematopoietic malignancies. Going a step further down the line, the multi-actions of SDCs in many levels make them appealing as potential pharmacological targets, either by targeting directly the tumor or indirectly the adjacent stroma.

## Tumor Microenvironment and HSPGs

Extracellular matrix (ECM) is a dynamic non-cellular network of macromolecules present within all tissues and organs. It is composed of a large collection of biochemically distinct components including collagens, elastin, fibronectin (FN), laminins, tenascin, vitronectin, thrombospondin, secreted protein acidic and rich in cysteine (SPARC), various proteoglycans (PGs), and hyaluronan (HA). These molecules of unique properties are able to provide the necessary mechanical structure for the cellular components but also contribute in several processes that are crucial for tissue morphogenesis, differentiation, and homeostasis (1, 2).

Cancer research has expanded and increasingly evolved over the years but there are still many unanswered questions due to the biological complexity of this pathological condition. One aspect of this complexity is attributed to the essential role of the stromal tissue in cancer progression. The tumor microenvironment is supported by a vascular network and contains several ECM molecules, fibroblasts, migratory immune cells, and neural elements, all within a milieu of cytokines and growth factors (3–5). The crosstalk between the cancer and the host stroma cells, via autocrine and paracrine complex mechanisms, recruits and activates the neighboring normal cells. This results in the re-organization of the stroma and it is often referred to as “reactive stroma” (6, 7). Both activated stromal and cancer cells exhibit a significant role in the re-organization of ECM in order to facilitate tumor cell growth, migration, and invasion (8, 9). PGs are among the key player components of stroma- and cancer-derived ECM. They interact with several structural components and matrix-associated proteins [growth factors, cytokines, and growth factor receptor (GF-R)] and during cancer progression their expression in tumor microenvironment is markedly modified (10). Heparan sulfate proteoglycans (HSPGs) can be found both at the cellular and extracellular matrices. The principal representatives of HSPGs are syndecans (SDCs), which have a single transmembrane domain; glypicans, which are linked to the outer plasma membrane by a glycosylphosphatidylinositol (GPI) anchor; and a group of secreted PGs, including perlecan, agrin, collagen XVIII, and the testican family (11, 12). They are present almost in all cell types and tissues and they act as regulators not only in normal but also in pathological conditions (13). Their regulatory role is attributed to their ability to collaborate with other matrix components contributing to basement membrane structural integrity and cell–cell as well as cell–matrix interactions. HSPGs, via their covalently bound heparan sulfate (HS) chains bind cytokines, chemokines, morphogens, and growth factors, serving also as signaling co-receptors (11, 14).

In the present review, we have addressed our attention to SDCs. There are four types of SDCs in mammals and probably in all vertebrates, whereas all the invertebrates and primitive chordates possess only one (15, 16). SDCs possess three distinct structural domains: the ectodomain with an N-terminal signal peptide and several sites for glycosaminoglycan attachment, which have a low sequence homology among the different types of SDCs, the highly conserved single transmembrane domain, and the short C-terminal cytoplasmic domain. Apart from HS chains, chondroitin sulfate (CS) GAG-attachment in SDC-1 and -3 is also reported (17, 18). The majority of cell types with the exception of the erythrocytes express at least one type of SDC and in several cases all four. Specifically, SDC-1, mainly expressed in epithelia as well as in some leukocytes, is responsible for mesenchyme condensation during development. On the other hand, its structural counterpart, SDC-3 is present in neural tissue and the developing musculoskeletal system. SDC-2 is distributed in mesenchymal tissues, fibroblasts, liver, and developing neural tissue, whereas SDC-4 is ubiquitously distributed (16, 19).

Syndecans are involved in a variety of complex signaling events through which they play regulatory roles for cell proliferation, differentiation, adhesion, and migration (8). Using mutated mice with altered HSPG core proteins expression as a tool, some important functions for SDCs have been identified. Results highlighted that the mice in which SDCs-1, -3, or -4 have been depleted, develop normally, are fertile, and have no obvious pathologies. Based on these observations, it is stated that the redundancy of an individual SDC has no critical role during development (20–23). Notably, SDC-1 null mice had epithelial cell migration defects, whereas SDC-3 null mice exhibited impaired radial migration and neural migration in development as well as partial resistance to obesity. A possible implication of SDC-3 has occurred in satellite cell maintenance, proliferation, and differentiation (24). On the other hand, SDC-4 is implicated in processes involving vascular defects in fetal placental labyrinth and poor angiogenic response in postnatal wound healing (14, 24). We should note at this point that SDC-2 mutants have not been reported so far, but it has been established that SDC-2 plays an important function in the angiogenic process (25). Moreover, Noguer et al. showed that SDC-2 impairs angiogenesis in human microvascular endothelial cells (26).

## Syndecans as Cell Surface Mediators in Cancer Biology

### Functions mediated by syndecans–ECM interactions

Syndecans exhibit a great variety in their localization and function and as a result, they are considered as key regulators of tumorigenesis and tumor progression (27). It is well established that SDCs may serve as biomarkers for early detection or severity of cancer. As presented in Table 1, they are expressed in a variety of cancer types, apart from SDC-3 that is not implicated in cancerous conditions. To note, SDCs possess diverse roles each time based on the type and stage of cancer, acting either as inhibitors or promoters of tumor progression (28, 29).

**Table 1:** **Overview of syndecans expression involving their origin, state, and processing enzymes**.

Syndecan member	Origin	Origin of shed syndecans	Origin of tumor microenvironment derived shed syndecans	Shedding enzymes
Syndecan-1	Epithelial cancers such as oral mucosa (30), uterine cervix (31, 32)	Cervical cancer (33) Myeloma cells (37–41) Pancreatic carcinoma (47) Hodgkin’s lymphoma (49) Breast cancer (33, 52, 53) Lymphoblastoid cells (57) Lung cancer (59) Bladder epithelial carcinoma cells (54)	Epithelial cells (34) Fibroblasts and endothelial cells (42–44)	MMP-9 (33) MMP-7 (45) MMP-2 (33) MT1-MMP and MT3-MMP (50) ADAM17 (54)
	Squamous cell carcinoma of neck, head, and lung (35, 36)			
	Laryngeal cancer (46)			
	Malignant mesothelioma (48)			
	Multiple myeloma (51)			
	Hepatocellular and colorectal carcinoma (55, 56)			
	Murine mammary carcinoma (58)			
	Ovarian cancer (60)			
	Breast cancer (61–63)			
	Pancreatic cancer (64)			
	Gastric cancer (65)			
	Hematological malignancies (66, 67)			
	Myeloma (66)			
	Gallbladder cancer (68)			
Syndecan-2	Melanoma (69)	Colon cancer (70)	Microvascular endothelial cells (71)	MMP-7 (70)
	Colon cancer (72)		Epithelial cells (73)	MMP-2
	Prostate cancer (74)			MMP-9 (71)
	Lung Lewis carcinoma (75)			
	Microvessels of mouse glioma cancer (71)			
	Esophageal squamous carcinoma (76)			
Syndecan-4	Breast cancer (63, 77)	Cervical cancer (33)	Stromal cells (10)	MMP-9 (33)
	Melanoma (78)	Lung epithelial carcinoma (54)	Lung epithelial cells (54)	ADAMTS-1 (79)
	Urinary bladder carcinoma (80)	Bladder epithelial carcinoma (54)		ADAMTS-4 (79)
	Osteosarcoma (81)			ADAM17 (54)
	Hematopoietic malignancy (83)			Plasmin and thrombin (82)
	Colon carcinoma (84, 85)			
	Testicular germ cell tumors (86)			

Several ECM macromolecules, such as FN, tenascin-C, collagen, thrombospondin, laminin, glycoproteins, etc., are documented to interact with SDCs. These specific interactions depend on the length diversity and extent of GAGs chains sulfation, which is actually different among cell types. On the other hand, the heparin-binding motifs of ECM macromolecules are responsible for SDCs–matrix interactions (19). Such close dynamic relations initiate signaling cascades, that in turn result in altered functional cellular properties. In highly metastatic colorectal cancer cells, SDC-2 is enhanced by stromal secreted FN promoting cell adhesion via simultaneous up-regulation of α2, β1-integrin, and FAK phosphorylation (87). Accordingly, high expression levels of SDC-4 and FN may be the underlying molecular alteration occurred in osteosarcoma, that lead to an aggressive phenotype (81). Furthermore, tenascin-C impairs the adhesive properties of FN by blocking SDC-4 co-receptor function in integrin signaling, thereby triggering tumor cell proliferation (88). Moreover, a peptide derived from tenascin-C, strongly activates β1-integrin functional activity through binding with SDC-4. These interactions lead to induced apoptosis selectively in hematopoietic tumor cells, which express adequate amounts of both integrin α4β1 (very late antigen-4, VLA-4) and SDC-4, driving FN-mediated effects (83). SDC-1 and -4 in collagen microenvironment create a complex interplay between integrin α2β1 and membrane type 1 metalloproteinase [MT1-matrix metalloproteinases (MMP)] in K-Ras mutated cells, promoting cell invasion and metastasis (89). Moreover, SDC-1 is essential for cell motility and invasion in collagen substrate via the modulation of RhoA and Rac activity in squamous cell carcinoma (90). Thrombospondin-1, a homotrimeric protein, is implicated in cancer cell adhesion, migration, and invasion as it activates the transforming growth factor beta (TGF-β) (91). On the top of that glioma cells secrete high levels of thrombospondin-1, which bind to ανβ3, α3β1 integrins, and SDC-1, participating in carcinoma cell motility and migration (92). Notably, SDC-1 expression in association with the high expression of thrombospondin-1 is mediated through NF-kB signaling effector (93).

### Syndecans as co-receptors for growth factors signaling

**Figure 1:**
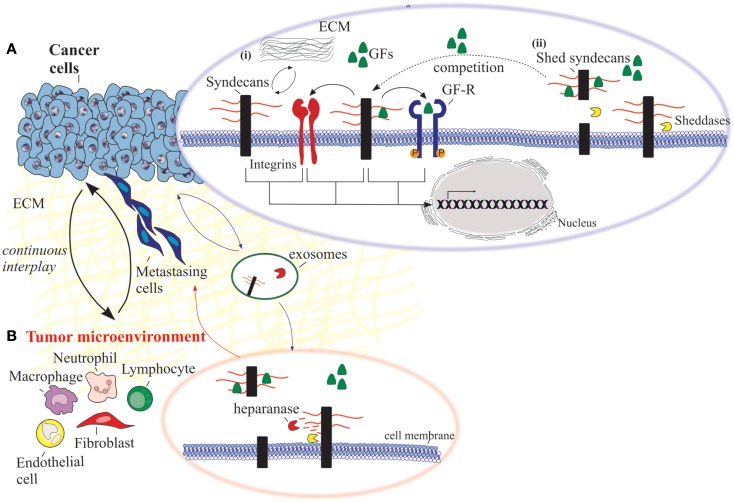
**An overview of the functional properties of syndecans in cancer cells and the adjacent tumor microenvironment**. **(A)** Cancer cells: (i) syndecans interact with various ECM macromolecules derived either from stromal cells or tumor cells. Such interactions lead to integrin-mediated altered functional properties such as cell proliferation, adhesion, migration, and invasion. On the other hand, transmembrane syndecans interact with growth factors (GFs) via their HS chains and subsequently act as co-receptors for the respective growth factor receptor (GF-R). In both cases, integrins can co-interact with these complexes and as a consequence to mediate different signaling pathways. (ii) Syndecan shedding is a process that involves the proteolytic cleavage of their ectodomain near the plasma membrane by sheddases. It is also reported that the shed syndecans compete with their transmembrane counterparts for soluble GFs. Shedding of syndecans contributes to cancer progression and especially to the crosstalk between the tumor cells and their host microenvironment. Exosomes, extracellular vesicles that are secreted in high amounts in tumors, retain both heparanase and syndecan-1 as cargo within exosomes and subsequently influence not only the behavior of the tumor microenvironment within the tumor niche and distant sites, but also the growth of the metastasizing cells. **(B)** Tumor microenvironment. Heparanase plays a distinct role in the shedding of syndecans by cleaving HS chains promoting the shedding via sheddases. This action results in induced tumor growth, angiogenesis and metastasis.

Syndecans are associated with several cell surface receptors and therefore regulate dynamically the binding of their adjacent ligands, forming active complexes. Some of the most common regulatory interactions of SDCs involve growth factors, integrins, and other signaling molecules (Figure 1). Fibroblast growth factors (FGFs)-mediated signaling via FGFRs regulate development and homeostasis. Characteristically, in breast carcinoma cells SDC-1 and SDC-4 regulate the formation of FGF-2/HSPG/FGFR-1 complex, indicating the importance of altered HS chains sulfation pattern during malignant conditions (94). Further investigation of the underlying mechanism indicated that SDC-1 and FGF-2, but not FGFR-1, share a common transport route and co-localize with heparanase in the nucleus at mesenchymal tumor cells (95). However, the effect of SDC-1 translocation on malignant cells, it has not yet clarified. On the other hand, it is clearly stated in the literature that HS chains of SDC-1 in premalignant epithelial cells interact with both FGFR-1 and -2 signaling complexes and this interaction is directly associated with the progression of malignancy (96). Moreover, in melanoma cell lines the expression of SDCs CS/HS chains appears to be modulated by FGF-2, that in turn facilitates signaling (97).

Vascular endothelial growth factor (VEGF) and insulin growth factor (IGF) are key regulators of vascular, organ, and neural development, but are also related to angiogenesis and cancer progression (98). In multiple myeloma, SDC-1 promotes endothelial cells proliferation and survival by modulating VEGF–VEGFR-2 signaling pathway (99). In addition, VEGF expression is significantly upregulated in cells expressing high levels of heparanase, leading to decreased nuclear SDC-1 and an aggressive tumor phenotype (100). In another line of studies on human breast carcinoma, SDC-1 ectodomain, but not SDC-4, regulates ανβ3 integrin–SDC signaling complex (77, 101). Although the engagement between SDC-1 and ανβ3 integrin occurs through the SDC-1 ectodomain, the activation is correlated with the cytoplasmic domain. On top of that, the extracellular interaction of SDC-1 with ανβ3 integrin promotes the docking of IGFR with SDC-1 ectodomain. The formation of IGFR/SDC-1/integrin complex seems to be a crucial regulator for integrin-mediated effect in tumor cell metastasis and tumor-induced angiogenesis (102). On the other hand, in mouse fibroblasts, constitutive association of SDC-1 with β5 integrin appears to be important for ανβ5-dependent signaling (103). According to the above data, a recent study presented that vascular endothelial-cadherin induces SDC-1 complex with IGFR and the subsequent crosstalk between ανβ3 and VEGFR-2 on endothelial cells during angiogenesis (104).

Epidermal growth factor (EGF), IGF, and platelet-derived growth factor-BB (PDGF-BB) are key players in several malignancies. In human mesothelioma, the presence of EGF, IGF, and PDGF-BB seems to regulate the levels of SDCs and these variations in the expression of SDCs are correlated with the growth factor signaling activation by an auto-regulatory loop mechanism (105). Histological immunostaining in patients with non-small cell lung carcinoma demonstrated that the expression of SDC-1 and EGFR is associated with patient survival (106). On the other hand, the low expression of SDC-1 stimulates the ovarian cancer cells invasion, mediated by heparin-binding EGF (HB-EGF) through a urokinase-independent mechanism (60). Moreover, EGF-family ligands are essential for multiple myeloma cell growth via binding with HSPGs and especially with SDC-1, which is abundantly expressed in this malignancy (107). It is also of great importance to point out the role of EGFR/IGFR signaling pathways in the expression of SDCs-2 and -4 in hormone-dependent breast cancer. The variations in the expression levels of SDCs, mediated through EGFR/IGFR signaling pathways, are correlated also with the migratory potential of breast cancer cells (108). Furthermore, the abundant PDGF-BB in cell microenvironment stimulates fibroblasts migration, inducing the level of SDC-4 (109). On the contrary, PDGF-mediated signaling in glioma cells initiates an induction of migration via a SDC-4-independent action (110). Reduced levels of SDC-4 in non-seminomatous germ cell tumors are related to their metastatic potential, whereas stromal staining of SDC-4 in testicular germ cell tumors is correlated with angiogenesis (86). SDC-2 binds to TGF-β via HS chains and promotes TGF-β signaling (111). In fibrosarcoma cells, TGF-β2 via Smad2 promotes cell adhesion and this function is directly modulated through SDC-2 (112). SDC-1 expression in breast cancer stroma fibroblasts regulates cell proliferation, angiogenesis, and cell motility. On the other hand, SDC-2 seems to exert antifibrotic effect by promoting caveolin-1-mediated TGF-βRI internalization and inhibiting TGF-β1 signaling (44, 61, 113).

## Shed Syndecans in Cancer Biology

As part of the normal turnover, SDCs undergo regulated proteolytic cleavage of their extracellular domain near the plasma membrane into the extracellular milieu. There it can be diffused away from the cell, be part of the ECM or remain soluble. In the soluble state, it can influence the surrounding or distal cells (37). This process is known as “shedding” and it happens under physiological conditions. However, shedding may be increased in response to stimuli (37, 114). The shed SDC not only downregulates signal transduction, but also converts the membrane-bound receptors into soluble effectors and/or antagonists. The remnant core protein at the cell surface loses its ability to bind ligands and can be further processed via intramembrane cleavage by the presenilin/γ-secretase complex (37).

### Syndecan sheddases

All mammalian SDC family members can be cleaved by extracellular proteases. The MMPs are zinc-dependent endopeptidases that play an important role at different stages of cancer. Several studies have shown that the expression of MMPs is upregulated during cancer progression, and it is directly associated with poor clinical prognosis (115). MMPs have been incriminated to shed the extracellular domain at membrane-proximal sites. For example, MMP-9 has been implicated in the stromal cell derived factor-1 (SDF-1)-mediated shedding of SDC-1 and SDC-4 in HeLa cells (33). It has been reported that matrilysin (MMP-7) as well as the membrane-associated metalloproteinases MT1-MMP and MT3-MMP are responsible for SDC-1 cleavage, whereas the gelatinases MMP-2 and -9 cleave SDCs-1 and -2 (50). The ADAMTS (disintegrin-like and metalloproteinase with thrombospondin motifs) family is also implicated in SDC shedding. It has been reported that both ADAMTS-1 and -4 cleave SDC-4 near the first GAG-attachment site that eventually results in decreased cell adhesion and promotion of cell migration (37, 79). In addition, ADAM17 has been reported to shed SDC-1 and -4, effect that is diminished following ADAM17 knockdown (54). It is also worth noticing that human SDC-4 is cleaved by the serine proteases plasmin and thrombin (82). Shedding can occur at different cases and different sites at the core protein of SDCs. Focused studies on the shedding mechanism will improve our knowledge regarding their potential implication in cell function. An overview of the documented shed SDCs and the respective sheddases in various cancer types is presented in Table 1.

### Regulatory mechanisms of syndecan shedding

Syndecan shedding is regulated by a variety of extracellular stimuli including growth factors (116), inflammatory chemokines (33, 45), bacterial virulence factors (117, 118), heparanase (39), insulin (119), oxidative stress (120), and others (37). In all cases, the cleavage occurs through direct action of the extracellular proteases, sheddases. The exact mechanism on the extracellular stimuli that influence them to mediate shedding is still unknown.

Several growth factors like EGF, TGF-α, HB-EGF, and amphiregulin induce the release of SDCs-1 and -4 in a concentration dependent manner (116). A step further, thrombin receptor and EGF-mediated shedding is associated with the activation of the ERK/MAPK pathway (116, 120). Although, it is reported that protein kinase C (PKC) activation by the phorbol 12-myristate 13-acetate (PMA) induces SDC-1 shedding in myeloma cells and that its inhibition reverts this effect, PKC signaling cascade is not involved in EGF- and thrombin-mediated shedding (37, 121). Furthermore, FGF-2 is reported to enhance the shedding of SDC-1 in pancreatic carcinoma cells. The overexpression and activation of matrilysin MMP-7 in these cells has been related with the FGF-2 stimulatory effect on MMP-7 along with SDC-1 shedding (47, 122).

Another aspect of shedding regulation involves the heparanase, an endoglycosidase that degrades HS chains. Increased expression of heparanase is highlighted throughout the literature in a great number of tumor types, associated with angiogenic and metastatic potential of tumor cells (123). Upregulation of heparanase expression or exogenous addition of recombinant heparanase to myeloma cells stimulates SDC-1 expression and shedding (39, 124). Interestingly, it is reported that the HS chains of SDCs are able to coordinate the ectodomain cleavage. According to Ramani et al., the attached HS chains of SDCs may suppress the activity of heparanase and subsequent its shedding (125).

Intracellular regulatory mechanisms play also important roles in an agonist-induced shedding. Notably, SDC-1 cytoplasmic domain interacts with the inactive GDP-bound form of Rab5, a small GTPase that regulates intracellular trafficking. Rab5 triggers the conversion from the inactive GDP-bound to active GTP-bound state in response to shedding promoters. According to Hayashida et al., a dominant negative form of Rab5, without the ability to switch between active and inactive state, inhibited the SDC-1 proteolytic cleavage, stating that Rab5 is a critical regulator of SDC-1 shedding acting as an on–off molecular switch (126). It has been also reported that intracellular trafficking is actually a key regulator of SDC-1 shedding (126). Many growth factors are also involved in SDC-2 shedding, since treatment of microvascular epithelial cells with EGF, FGF-2, or VEGF induced this processing (71). It is also likely that SDC-1 shedding upregulates the SDC-2 synthesis affecting positively the proliferation of the cancer cells (127).

### Functional insights of syndecan shedding

The effects of SDCs shed ectodomain have been incriminated in several steps of cancer progression. Studies have pinpointed soluble SDC-1 ectodomain in the serum of lung cancer patients (59), Hodgkin’s lymphoma patients (49), and myeloma patients as well as within the ECM of the myeloma microenvironment (40, 41). In breast cancer, it is stated that the shed SDC-1 is derived largely from the host fibroblasts of the tumor microenvironment. To continue, based on studies in ARH-77 human lymphoblastoid cells, shed SDC-1 is established to promote tumor growth and progression *in vivo*, mediated by the crosstalk between tumor and host cells (57). Moreover, MT1-MMP-induced SDC-1 shedding inhibits cell migration in HEK293T cells (50) and cell proliferation in T47D breast carcinoma cells (52).

The significance of SDC shedding in malignancies is well documented in myeloma cells. As stated above, soluble SDC-1 is present at high levels in the serum of myeloma patients, promoting the growth of myeloma tumors *in vivo*. This observation renders shed SDC-1 as an indicator of poor prognosis in myeloma (38, 66, 123, 128). In addition, heparanase seems to play a distinct role in the shedding of SDCs in myeloma. It mediates shedding by cleaving the less sulfated regions along the HS chains creating fragments of 10–20 residues, promoting tumor growth, angiogenesis, and metastasis (129, 130). It is also documented that heparanase initiates the growth of myeloma cells and promotes bone metastasis by increasing the size of blood vessels within the tumor (37, 131–133). The basic idea is that the shed SDC-1 binds to growth factors derived from the tumor and concentrates them in the tumor microenvironment, promoting their signaling activity (Figure 1). On the other hand, upregulation of heparanase in the tumor microenvironment leads to elevated active levels of the intracellular effector ERK (p-ERK), and in turn increased expression of VEGF and MMP-9 (123). These effects are mediated not only from the high phosphorylation of ERK, but also due to the diminished levels of SDC-1 in the nucleus, leading to increased levels of acetylated histone H3 and eventually facilitating the transcription of VEGF and MMP-9 (100). As a result, MMP-9 cleaves SDC-1 from the cell surface and therefore interacts with growth factors like hepatocyte growth factor (HGF) and VEGF, whose expression is already stimulated by heparanase (134). Then, the “loaded” with growth factors shed SDC-1 binds to ECM macromolecules, such as FN and collagen, rendering these growth factors available in the tumor microenvironment even in distal sites. As a consequence, shed SDC-1 mediates the signaling of bound growth factors leading to a strong downstream signaling to host cells, triggering the microenvironment to support aggressive tumor growth (57).

Another aspect of heparanase action involves its cooperation with SDC-1 that regulates the biogenesis and function of the exosomes. These lipid bilayer bound extracellular vesicles are very aggressive especially when they are secreted in high amounts in tumors. Their cargo including proteins, mRNA, and miRNA, are of outmost importance for intracellular communication within tumor and host cells (135, 136). In many cases, exosomes derived from tumor cells are reported to promote angiogenesis (137), metastasis (138, 139), and immune evasion (140). In addition, both heparanase and SDC-1 are retained as cargo within exosomes and subsequently influence not only the behavior of the tumor microenvironment within the tumor niche and distant sites, but also the growth of the metastasizing cells (Figure 1) (57).

Similar to SDC-1, there is evidence indicating that the shed ectodomains of SDC-2 and -4 increase angiogenesis. Studies have shown that recombinant shed SDC-2 is cleaved by the MMP-7 in colon cancer but the consequences of this effect are not yet determined (141). Moreover, secreted ADAMTS-1 cleaves the ectodomain of SDC-4, affecting cytoskeleton organization and adhesion, enhancing angiogenesis and migration (79).

Shed SDCs are also in position to eliminate the inhibitory soluble factors. Shed SDC-1 facilitates the resolution of neutrophilic inflammation by inducing the clearance of proinflammatory CXC cytokines. In other words, shed SDCs may promote cancer also by sequestrating inhibitory signals (142).

Importantly, the soluble SDC ectodomain may serve as new soluble effectors and even compete with intact cell membrane SDCs for extracellular ligands in the host microenvironment (37, 143). In addition, there are several studies indicating that shed SDCs might act as ligands to induce gene expression. In particular, shed SDC-1 expression is reported to regulate the expression of TIMP metallopeptidase inhibitor 1 (TIMP-1), urokinase plasminogen activator receptor (uPAR), and E-cadherin in breast cancer cells coordinating their invasiveness (53). Finally there are emerging evidence indicating that HSPG shedding can down-regulate HSPG-dependent functions by binding the appropriate HS ligands and making them accessible for internalization. Based on this observation, shed SDCs may actually have a role as extracellular chaperones that transfer ligands to cell surface HSPGs on neighboring cells based on the paracrine stimulation (144).

Finally, it is worth noticing that shed SDCs have also been reported to have anti-tumorigenic effects in several cases. To state, the membrane-bound SDC-1 promotes proliferation and inhibits invasiveness of MCF-7 breast cancer cells, whereas the soluble form has the exact opposite effect (53). Moreover shed SDC-1 inhibits alveolar epithelial wound healing, promotes fibrogenesis (145), and decreases invasion of TIMP-1-sensitive breast cancer cell invasion (53, 121).

## Potential Syndecan-Based Pharmacological Approaches in Cancer Treatment

As the knowledge of HSPGs role on cancer progression and development is accumulating, the perspective to use SDCs in therapeutics is becoming more and more appealing. An overview of SDCs-based therapeutic targeting is summarized in Table 2. The treatment with already existed pharmaceutical formulations in several *in vitro* and *in vivo* biological systems, suggests that they can regulate the expression levels of SDCs, thus inhibiting their carcinogenic potential. According to that notion, the third generation bisphosphonate, zoledronate (zoledronic acid, Zometa^®^) is shown to down-regulate the expression levels of SDC-1 and -2, in contrast with the upregulation of SDC-4 in human breast cancer cells with different metastatic potentials (63). This effect is associated with the inhibition of cell growth, migration, adhesion, and invasion in correlation with the diminished levels of ανβ3, ανβ5, and α5β1 integrins (63). Similar mode of action has the specific tyrosine kinase inhibitor imatinib (Glivec^®^), which targets PDGFRs, c-Kit and Bcr-Abl. It exerts a significant inhibitory effect on the expression of SDCs-2 and -4 on PDGF-BB-treated breast cancer cells, leading to suppressed cell growth ability, migration, and invasion (146). Also, Nimesulide a worldwide known non-steroidal anti-inflammatory drug, with specific action on cyclooxygenase (COX-2) inhibits the expression of SDC-1 in primary effusion lymphoma and blocks its anti-tumorigenic action (147).

**Table 2:** **Overview of syndecans (SDCs)-based therapeutic targeting**.

Syndecan targeting therapies	Name	Biological effect
Antibodies	Anti-syndecan-1 monoclonal antibody, nBT062 (148)	Inhibition of multiple myeloma cell adhesion
	Iodine-131-labeled anti-syndecan-1 antibody, B-B4 (149, 150)	Radioimmunotherapy in multiple myeloma and triple-negative breast cancer
	Anti-syndecan-1 antibody, OC-46F2 (151)	Reduction of syndecan-1/VEGFR-2 binding
Enzymes inhibitors	MMPs inhibitors (152, 153)	Inhibition of syndecans shedding in multiple myeloma and pancreatic cancer
	SST0001 heparanase inhibitors (154)	
Biomolecules as inhibitors	Synstatin (152)	Inhibition of the formation of syndecan-1/ανβ3, ανβ5 integrins/IGFR complex
	All-trans retinoic acids (153)	Inhibition of syndecans shedding in lung cancer
Synthetic inhibitors	STI571, specific tyrosine kinase inhibitor, imatinib (Glivec^®^) (146)	Inhibition of cell growth and migration by regulating syndecans expression levels
	Third generation bisphosphonate, zoledronate (zoledronic acid, Zometa^®^) (63)	

Recent studies focus on exploring therapeutically approaches that are associated with SDCs ectodomain. As a result monoclonal antibodies or peptides, which target specifically extracellular domain of SDCs, have been evaluated. OC-46F2, a fully human anti-SDC-1 recombinant antibody, reduces SDC-1/VEGFR-2 distribution in tumor microenvironment, resulting in suppressed vascular maturation and tumor growth in melanoma and ovarian experimental model (151). It has also been suggested that antibodies against SDCs, especially SDC-1 and -4, are able to inhibit the dynamic relations between SDCs and cytokines leading to potential treatment of hepatocellular carcinoma (155, 156). To continue, a murine/human chimeric anti-SDC-1 monoclonal antibody, nBT062, conjugated with highly cytotoxic maytansinoid derivatives was introduced. The nBT062-maytansinoid conjugation was reported to drive targeted maytansinoid-induced cytotoxicity in multiple myeloma, blocking cell adhesion to bone marrow stromal cells. Moreover, these conjugations inhibit multiple myeloma tumor growth *in vivo* and prolong host survival in both xenograft mouse models of human multiple myeloma and SCID-hu mouse model (148). In addition, B-B4 (iodine-131-labeled anti-SDC-1 antibody) was administrated to myeloma patients with success, promoting the notion of targeted radioimmunotherapy (RIT) (149). Interestingly, recent studies indicate the importance of B-B4 antibody not only in multiple myeloma but also in triple-negative breast cancer in combination with immune-PET imaging and RIT (150). Another approach in SDC targeting involves the use of small peptides. For example, Synstatin was developed to the sequence between 82 and 130 amino acids of SDC-1 ectodomain. In detail, this peptide antagonizes SDC-1 domain, responsible for capturing and activating ανβ3 or ανβ5 integrins and IGF-IR. To note, Synstatin’s action prevents the formation of the receptor complex, and in turn blocks tumor-induced angiogenesis and metastasis mediated by the initial complex (152).

Considering the significant role of shed SDCs, their pharmacological potential was investigated in several studies targeting indirectly their actions. It is noted that myeloma and pancreatic chemotherapeutic drugs tend to induce accumulation of shed SDC-1 exactly as benzo(α)pyrene does in lung cancer. To avoid such tumor initiating effect, the use of metalloproteinase inhibitors in combination with chemotherapy and all-trans retinoic acid was suggested (153, 157). Another strategy to accomplish shedding inhibition involves the use of SST0001, a non-anticoagulant heparin with anti-heparanase activity, whose use diminishes the heparanase-induced SDC-1 shedding. In addition, the combination of SST0001 with dexamethasone, blocks tumor growth *in vivo* presumably through dual targeting of the tumor itself as well as its microenvironment (154). A recent study in multiple myeloma highlighted that targeting HS expression, through knockdown of EXT1, in combination with exposure to lenalidomide or bortezomib results in inhibition of cell growth (158).

Based on the ability of SDCs to act as endocytosis receptors, SDCs have been used for viral and non-viral scaffolds that deliver nucleic acids for gene therapy. Specifically, lipoplexes and nucleic acid polyplexes before entering into the cell bind on SDCs clusters in actin-rich plasma membrane extensions, and therefore are internalized driven by the action of the cytoskeleton retrograde flow (159). Polyethyleneimine (PEI)–DNA conjugates represent a drug delivery mechanism according to which SDC-1 is required for the successful gene transfer whereas SDC-2 inhibits this process (160).

## Concluding Remarks

Syndecans represent an ongoing field of investigation, attempting to elucidate their regulatory roles in normal and pathological conditions. Multiple roles of SDCs in cancer progression are documented, implicating them in diagnosis, progression, and even the treatment of different types of cancer. The dynamic potential of these HSPGs is vast, considering their transmembrane localization, their ability to promote signal transduction through interactions with a plethora of ligands, the interplay between tumor and stroma cells and last but certainly not least their ability to be cleaved upon different stimulations. An overview of the major reported mediated effects of SDCs (transmembrane and shed) are depicted in Figure 1. These molecules have strong implications in cancer biology in membrane-bound state and also in their soluble state, it is a fact that SDCs can function in different ways, different stages of cancer, and to regulate tumorigenic mechanisms. The promising *in vitro* and *in vivo* results indicate the feasibility of targeting SDCs as a potential cancer treatment strategy. However, in order this concept to be shaped, further preclinical studies and well-designed clinical trials are necessary. Anticancer drugs will be probably produced through pharmacological targeting of shed SDCs in combination with agents responsible for inhibition of signal transduction and epigenetics. Taking into consideration the SDCs-mediated biological actions during early stage and progression of cancer, it is plausible to suggest that members of the SDCs family may be a potential tool for disease diagnosis and prognosis as well as candidates for designing novel therapeutic approaches against cancer progression.

## Conflict of Interest Statement

The authors declare that the research was conducted in the absence of any commercial or financial relationships that could be construed as a potential conflict of interest.
